# Appropriate neck circumference and waist-to-height ratio cut-off points as predictors of obesity and cardiovascular risk in adolescents

**DOI:** 10.11606/s1518-8787.2023057004349

**Published:** 2023-03-30

**Authors:** Wyllyane Rayana Chaves Carvalho, Ana Karina Teixeira da Cunha França, Alcione Miranda dos Santos, Luana Lopes Padilha, Eduarda Gomes Bogea

**Affiliations:** I Universidade Federal do Maranhão Programa de Pós Graduação em Saúde Coletiva São Luís MA Brasil Universidade Federal do Maranhão. Programa de Pós Graduação em Saúde Coletiva. São Luís, MA, Brasil; II Universidade Federal do Maranhão Departamento de Saúde Pública São Luís MA Brasil Universidade Federal do Maranhão. Departamento de Saúde Pública. São Luís, MA, Brasil

**Keywords:** Adolescent, Waist-Height Ratio, Neck, Anthropometry, Heart Disease Risk Factors, Obesity

## Abstract

**OBJECTIVE:**

To determine neck circumference (NC) and waist-to-height ratio (WHtR) cut-off points as predictors of obesity and cardiovascular risk in adolescents.

**METHODS:**

Cross-sectional study developed with a subsample of 634 adolescents aged 18 and 19 years belonging to the third phase of the “RPS” cohort (Ribeirão Preto, Pelotas and São Luís) carried out in 2016. The area under the ROC curve (AUC) was identified to assess the predictive capacity of NC and WHtR in relation to the percentage of body fat (%BF), obtained by air displacement plethysmography (ADP), and the cardiovascular risk estimated by the Pathobiological Determinants of Atherosclerosis in Youth (PDAY).

**RESULTS:**

The prevalence of obesity by %BF was 7.6% in males and 39.4% in females (p-value <0.001), and the high PDAY risk was 13.8% and 10.9%, respectively. For males, NC cut-off point was 44.0 cm and the AUCs were 0.70 (95%CI 0.58-0.83) to predict obesity and 0.71 (95%CI 0.62-0.80) to predict high cardiovascular risk; for females, NC cut-off point was 40 cm and the AUCs were 0.75 (95%CI 0.69-0.80) and 0.63 (95%CI 0.53-0.73), respectively. WHtR cut-off point was 0.50 for both sexes; for males, the AUCs to predict obesity and high risk according to PDAY were 0.90 (95%CI 0.80-0.99) and 0.73 (95%CI 0.63-0.82), respectively; for females, they were 0.87 (95%CI 0.83-0.90) and 0.55 (95%CI 0.45-0.65), respectively.

**CONCLUSION:**

WHtR and NC are good discriminators to assess obesity and cardiovascular risk in adolescents, especially in males.

## INTRODUCTION

In the last decades, overweight and obesity have shown substantial growth and are cause of concern worldwide, since have relevant outcomes on public health^1^.

Most critical periods for the development of excess body fat occur in early childhood and adolescence; however, the accumulation of fat in adolescence tends to remain in adulthood^2^.

It is a consensus in the literature that obesity, especially central obesity, predisposes individuals to chronic noncommunicable diseases (NCDs), which can be explained by being related to specific metabolic conditions that favor the occurrence of dyslipidemia, arterial hypertension, insulin resistance, and diabetes^3,4^.

Those conditions have become a worldwide epidemic^5^. Faced with this challenge, the World Health Organization (WHO) established as global goal for 2025 the reduction of premature mortality from NCDs by 25% and, for this this to occur, one of the axes is to stop the increase in obesity prevalence^6^. In this perspective, it is important to carry out the diagnosis of obesity based on the use of simple and accurate instruments to assess excess body fat.

Several studies propose anthropometric indicators to determine the association between obesity and cardiovascular risk. Most of them used traditional indicators and methods, including Body Mass Index (BMI) and Waist Circumference (WC), in order to compare the performance of these indicators in detecting general body fat and cardiovascular risk^7-9^.

Recently, other indicators began being studied, including waist-to-height ratio (WHtR) and neck circumference (NC)^9^. WHtR is considered an important tool for identifying body fat and risk of cardiovascular diseases^5,13,14^, and has gained prominence in population studies in different age groups^10,11^. Furthermore, when compared to other indicators, the determination of a single WHtR cut-off point value is suggested as an advantage as a good anthropometric indicator in public health^12^. In national and international studies, there is a small variation in WHtR cut-off points with the objective of predicting obesity and cardiovascular risk in children, adolescents and adults, although the determined values are close or equal to 0.50^9,14-17^.

With regard to NC, it should be noted that the neck fat is essentially subcutaneous^7^, which would explain its correlation with cardiovascular risk and insulin resistance^18^, as there is greater lipolytic activity in this fat compartment, especially in obese individuals^19^. Although new, this indicator has the following advantages: good performance in determining obesity in childhood and adolescence; quick and simple measurement^7^, and lack of influence from postprandial abdominal distension or respiratory movements^20^.

However, there are few studies that have evaluated its capacity to predict obesity and cardiovascular risk, especially in adolescents.

Considering the importance of identifying the predictive capacity – based on methods that are considered the gold standard – of anthropometric indicators to identify obesity and cardiovascular risk in adolescents and that can be used in health care, this study aimed to determine NC and WHtR cut-off points using air displacement plethysmography (ADP) to predict obesity and cardiovascular risk in adolescents.

## METHODS

### Study Design and Sample

Cross-sectional study carried out with data from the RPS Birth Cohort Consortium produced by three Brazilian cities (Ribeirão Preto, Pelotas and São Luís) and approved by the University Hospital Research Ethics Committee of the Federal University of Maranhão (CEP/HU-UFMA) (Opinion No. 1.302.489).

Participants in the cohort from the city of São Luís were evaluated in three stages of life: 1^st^phase – birth; 2^nd^ phase – childhood (7 to 9 years old), and 3^rd^ phase – adolescence (18 and 19 years old). The detailed methodology can be found in the study by Bragança et al.^21^. For this work, only data from the 3^rd^ phase were used.

The 3^rd^ phase of the cohort was carried out in 2016, with individuals aged 18 and 19 years, and aimed to assess nutritional outcomes, chronic diseases, mental health, and human capital. In this phase, 2,515 adolescents were evaluated, 654 belonging to the birth phase (1^st^phase) and 1,861 adolescents born in 1997 in São Luís, MA, who were included to increase the sample power and predict future cohort losses. Teenagers were included in the 3^rd^phase from selection at the four Army Recruiting Centers on the island of São Luís (MA); at high schools based on data from the 2014 school census, and at universities. These adolescents were submitted to the same tests and instruments applied to adolescents in the birth phase. A questionnaire was also applied to mothers to collect perinatal data.

**Figure 1:**
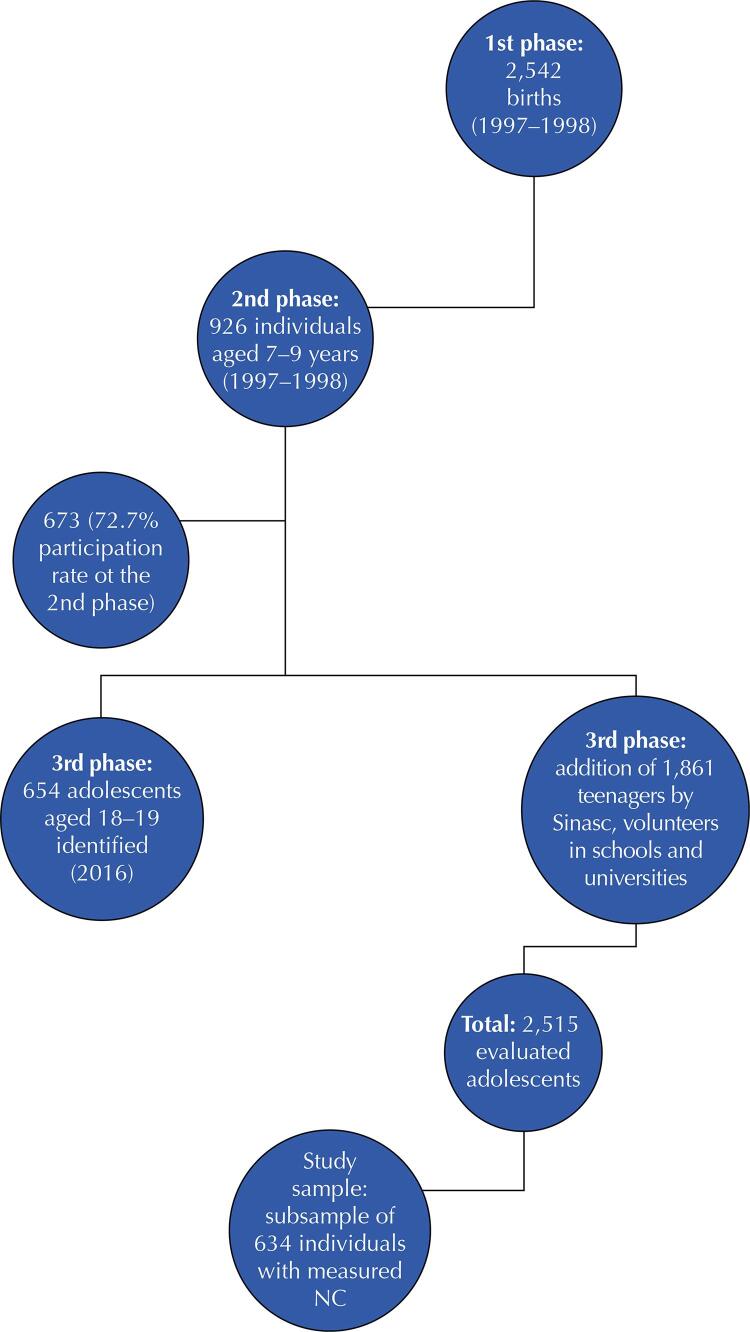
Flowchart of the study sample.

Regarding the 2,515 adolescents evaluated in the 3^rd^ phase, only those who had their NC, WHtR and (%BF) measured were included in this study. After applying these criteria, 634 adolescents were eligible to compose the final sample. Figure 1 illustrates the study sampling plan.

Sinasc: Live Birth Information System; CP: neck circumference.

### Data Collection and Analysis

Data were obtained by a trained team by applying questionnaires and using equipment, and recorded on the Research Electronic Data Capture (Redcap^®^)^22^ software.

The following socioeconomic and demographic data were used: sex (male and female); age (18 and 19 years old); socioeconomic class, according to Economic Classification in Brazil (CEB) (A/B, C and D/E); currently studying (‘yes’ and ‘no’); currently working (‘yes’ and ‘no’); self-declared skin color (black, brown, and white); schooling (elementary school, high school, high school technical education, technical education, higher education in progress, pre-college entrance exam courses, EJA/PEJA), and smoking (‘yes’ and ‘no’).

The anthropometric data of interest were body weight, height, WC, and NC. To measure weight (in kg), the ADP scale was used, on which the adolescents were positioned standing, in the center of the equipment, barefoot, wearing tight-fitting Lycra clothes, with gym top for females and shorts for both sexes. Height (cm) was measured using an Alturexata^®^ stadiometer, with the adolescents barefoot and standing in the center of the equipment, with hands along the body, upright posture, face facing forward, in the Frankfurt Plane, and observing a fixed point. NC and WC (in cm) were obtained from the three-dimensional body image using a 3-Dimensional Photonic Scanner (3DPS-([TC] Labs, Cary, United States). Waist-to-height ratio was calculated using the ratio between WC and height.

BMI was used to assess the adequacy of weight to height. This was obtained through the ratio of body weight (kg) to height (m^2^), classified in Z-score, according to sex and age. The following criteria were used: underweight (< Z-scores -2); normal weight (≥ Z-Scores -2 and < Z-Scores +1); overweight (≥ Z-score +1 and < Z-score +2), and obesity (≥ Z-score +2)^23^. NC and WHtR were used to evaluate abdominal fat.

The PDA technique was used to verify body adiposity using the Cosmed Bod Pod^®^ Gold Standard device (Rome, Italy). At the time of the test, the adolescents were wearing the same clothes as in the anthropometric measurements, and a cap was provided to compress their hair during ADP. The plethysmograph was calibrated daily with a known 50-liter volume. Based on the measured body volume and body mass, the device calculated body density, which was used in Siri equation to determine the adolescents’ fat mass ^24^.

Regarding body fat percentage, the adolescents were classified as obese (≥ 25% for males and ≥ 30% for females) and non-obese (< 25% for males and < 30% for females) by Williams et al.^25^.

Blood pressure was checked using the oscillometric method with an Omron^®^ HEM-7221NT automatic blood pressure monitor. Cuffs of an appropriate size regarding arm circumference were used and the mean of the three systolic and diastolic blood pressure measurements taken was considered, after one minute of rest, in the sitting position, with the dominant arm resting on a support so that the radial artery was at the same level as the heart.

The biochemical markers used were postprandial glycemia, total, HDL and LDL cholesterol levels, measured from the individuals’ blood analysis.

The serum was separated and stored in an Eppendorf at -80°C until analysis. At the time of blood collection, the adolescents were not fasting and were not asked about the time of their last meal. The samples were analyzed in the laboratory of the School of Dentistry of the Federal University of Maranhão (UFMA), using the Milliplex MAP Human Cytokine Kit, manufactured by Merck (Darmestádio, Germany).

Cardiovascular risk was assessed using the Pathobiological Determinants of Atherosclerosis in Youth (PDAY), which is a global risk algorithm with multiple cardiovascular risk factors, and has the advantage of estimating the probability of early atherosclerotic lesions in adolescents and young adults^26^. This was developed based on the Framingham Risk Score (FRS) and establishes the premise that risk factors for cardiovascular disease are associated – decades before the cardiovascular outcome – with the initial and advanced phases of atherosclerotic lesions during adolescence and early adulthood. Risk stratification by PDAY is obtained by adding the values attributed to modifiable factors such as non-HDL cholesterol, HDL cholesterol, smoking, blood pressure, BMI, fasting glucose (FG) and glycosylated hemoglobin (HBA1c), as well as demographic factors (age, sex). If the result of the sum obtained is greater than zero, the probability of atherosclerotic lesions is estimated; therefore, cardiovascular risk^27,28^.

Thus, considering these stratifications, PDAY was obtained in this study from the variables and their respective scores: age (in years, from 10-19 = 0; 20-24 = 5; 25-29 = 10; 30-34 = 15 points); sex (male = 0; female = -1 point); non-HDL cholesterol (in mg/dL, < 130 = 0; 130-159 = 2; 160-189 = 4; 190-219 = 6; ≥ 220 = 8 points); HDL cholesterol (in mg/dL, < 40 = 1; 40-59 = 0; ≥ 60 = -1 point); smoking (no = 0; yes = 1 point); blood pressure (normal = 0; high = 4); obesity (assessed by BMI, non-obese = 0 and obese = 6 for males; non-obese and obese = 0 for females), and hyperglycemia (postprandial glucose < 140mg/dL = 0 and glucose in fasting ≥ 140mg/dL = 5 points). From the sum of the scores of each variable, cardiovascular risk was classified as low (score = 0), intermediate (score ≥ 1 and ≤ 4), and high (score ≥ 5 points).

### Statiscal Analysis

Sociodemographic, nutritional and cardiovascular risk variables were described using absolute and relative frequencies. Only the age variable was described using mean and standard deviation. To verify the normality of the age variable, the coefficient of asymmetry and coefficient of kurtosis were calculated and Shapiro-Wilk test was performed.

The Receiver Operating Characteristic (ROC) curve was used to analyze the predictive validity of NC and WHtR in discriminating obese adolescents, in relation to the %BF obtained by ADP, and with high cardiovascular risk, in relation to PDAY. AUC and confidence intervals were determined, and NC and WHtR values with the best balance between sensitivity and specificity were identified.

The ROC curve is a graphical method used to evaluate, organize, and select diagnostic and/or prediction systems. AUC describes the probability of identifying correctly individuals who are true positives and those who are not. These values are statistically significant when the lower limit of the 95%CI is greater than 0.50. AUC values are considered excellent when between 0.90–1.00; good, between 0.80–0.90; reasonable, between 0.70–0.80, and poor, between 0.60–0.7029 ^29^.

Data were exported from Redcap^®^ for analysis on STATA^®^ version 14 software. A 5% significance level and a 95% confidence interval (95%CI) were adopted.

## RESULTS

A total of 634 adolescents were evaluated: the mean age was 18.5±0.5 years and the majority was female (54.4%), single (98.0%), self-declared brown (61.4%), and belonging to class C (43.7%). Of these, 35.2% reported taking pre-college entrance exam courses or being in higher education (Table 1).

**Table 1:** Socioeconomic and demographic characteristics of adolescents in the RPS birth cohort (Third phase), São Luís, Maranhão, Brazil, 2016.

Variables	n	%
Sex		
Male	289	45.6
Female	345	54.4
Marital status		
Single	621	98.0
Common-law marriage	13	2.0
Skin-color		
White	133	21.0
Black	106	16.7
Brown	389	61.4
Yellow	2	0.3
Ignored	4	0.6
Schooling		
Elementary School	1	0.2
Technical Education	30	4.7
Higher education in progress	223	35.2
Pre-college entrance exam courses	223	35.2
EJA/PEJA^a^	11	1.7
Ignored	146	23.0
Socioeconomic class^b^		
A/B	203	32.0
C	277	43.7
D/E	98	15.5
Ignored	56	8.8
Total	634	100.0

^a^ EJA/PEJA: Youth and Adult Education Program.

^b^ According to Economic Classification in Brazil (CEB).

According to the BMI diagnosis, obesity was noted only in males: 3.8%. Through ADP, there was an obesity prevalence of 7.6% in males and 39.4% in females (p-value < 0.001). The prevalence of adolescents with high cardiovascular risk, assessed by PDAY, was higher in males (13.8% versus 10.9%; p<0.001) (Table 2).

**Table 2:** Nutritional status and cardiovascular risk of adolescents in the RPS birth cohort (Third phase), São Luís, Maranhão, Brazil, 2016.

Variables	Total n (%)	Male n (%)	Female n (%)	p-value
BMI				0.001
Underweight	26 (4.1)	10 (3.5)	16 (4.6)	
Normal Weight	505 (79.7)	235 (81.3)	270 (78.3)	
Overweight	92 (14.5)	33 (11.4)	59 (17.1)	
Obesity	11 (1.7)	11 (3.8)	0 (0.0)	
%BF, by ADP				< 0.001
Not obese	476 (75.1)	267 (92.4)	209 (60.6)	
Obese	158 (24.9)	22 (7.6)	136 (39.4)	
Cardiovascular risk, by PDAY				< 0.001
Low	398 (61.4)	149 (51.4)	249 (69.6)	
Intermediate	171 (26.4)	101 (34.8)	70 (19.6)	
High	79 (12.2)	40 (13.8)	39 (10.9)	

BMI: body mass index. %BF: percentage of body fat. ADP: air displacement plethysmography. PDAY: Pathobiological Determinants of Atherosclerosis in Youth.

**Figure 2:**
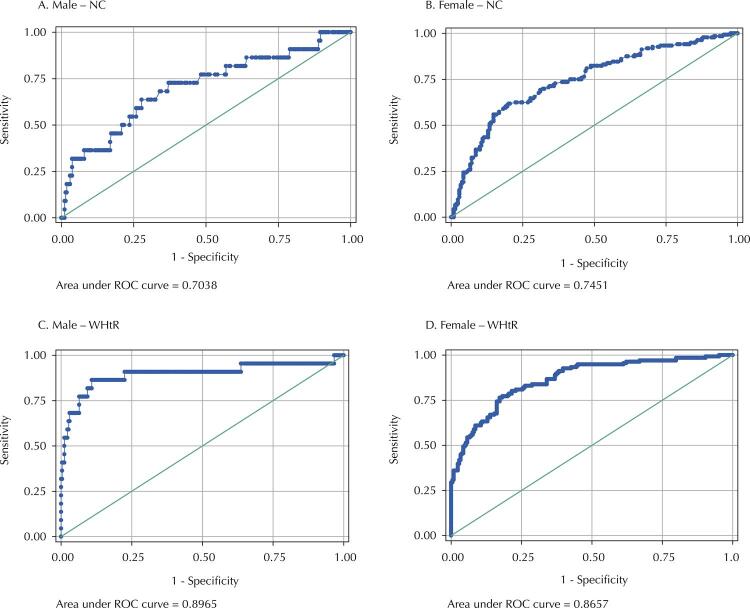
Area under the Roc curve and 95%CI of NC and WHtR with obesity, assessed by air displacement plethysmography (ADP), in adolescents of both sexes in the birth cohort RPS (third phase), São Luís, Maranhão, Brazil, 2016.

NC and WHtR AUC-ROC for prediction of obesity are shown in Figure 2 and Table 3. For males, NC AUC was 0.70 (95%CI 0.58–0.83) and WHtR AUC was 0.90 (95%CI 0.80–0.99), while, for females, NC AUC was was 0.75 (95%CI 0.69–0.80) and WHtR AUC was 0.87 (95%CI 0.83–0.90). The anthropometric indicators evaluated showed a statistically significant predictive capacity to identify obese individuals in both sexes.

**Table 3:** Sensitivity and specificity of the cutoff points of NC and WHtR in relation to the measure of high body adiposity obtained by the ADP, and the cardiovascular risk assessed by the PDAY, of adolescents from the birth cohort RPS(third phase), São Luís, Maranhão, Brazil, 2016.

Variables	AUC	CI95%	NC	Sensitivity	Specificity
**Male sex**					
%BF					
NC (cm)	0.70	0.58-0.83	44.0	68.2%	65.9%
WHtR	0.90	0.80-0.99	0.50	90.9%	75.3%
PDAY					
NC (cm)	0.71	0.62-0.80	44.0	63.2%	63.8%
WHtR	0.73	0.63-0.82	0.50	63.2%	72.5%
**Female sex**					
%BF					
NC (cm)	0.75	0.69-0.80	40.0	64.7%	72.2%
WHtR	0.87	0.83-0.90	0.50	78.7%	79.4%
PDAY					
NC (cm)	0.63	0.53-0.73	40.0	61.8%	59.2%
WHtR	0.55	0.45-0.65	0.50	50.0%	55.9%

ADP: air displacement plethysmography. %BF: percentage of body fat. AUC: area under the Roc curve. CI: confidence interval. NC: neck circumference. WHtR: waist-to-height ratio. PDAY: Pathobiological Determinants of Atherosclerosis in Youth.

**Figure 3:**
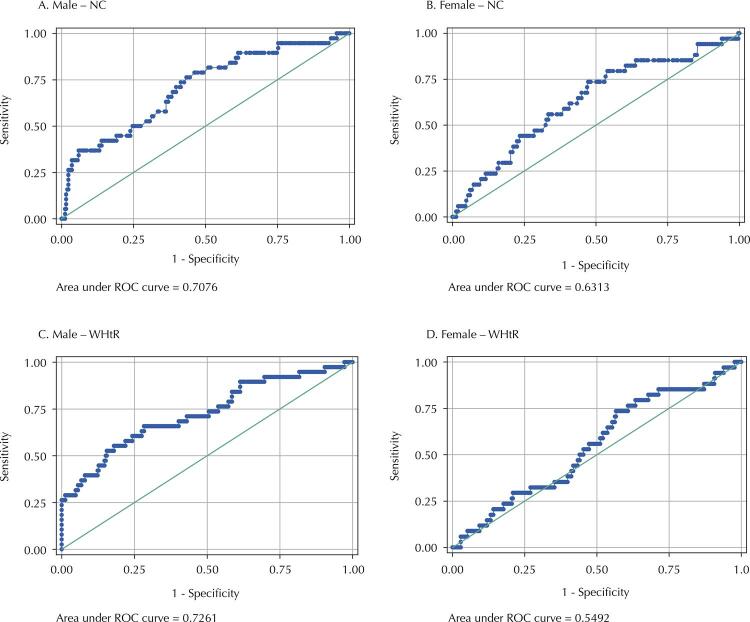
Area under the Roc curve and 95%CI of NC and WHtR with cardiovascular risk, assessed by Pathobiological Determinants of Atherosclerosis in Youth (PDAY), in adolescents of both sexes of the birth cohort RPS (third phase), São Luís, Maranhão, Brazil, 2016.

Figure 3 and Table 3 show NC and WHtR AUC-ROC to predict high cardiovascular risk. For males, NC AUC was 0.71 (95%CI 0.62–0.80) and WHtR AUC was 0.73 (95%CI 0.63–0.82), while, for females, NC AUC was was 0.63 (95%CI 0.53–0.73) and WHtR AUC was 0.55 (95%CI 0.45–0.65). Only WHtR did not show a statistically significant predictive capacity to identify adolescents with high cardiovascular risk.

To predict obesity and high cardiovascular risk, one identified the NC cut-off points of 40.0 cm for females (64.7% sensitivity and 72.2% specificity for obesity, respectively; and 61.8 % and 59.2% for cardiovascular risk, respectively) and 44.0 cm for males (68.2% sensitivity and 65.9% specificity for obesity, respectively; and 63.2% sensitivity and 63 .8% specificity for cardiovascular risk, respectively). A WHtR cut-off point of 0.50 was identified for both sexes to predict obesity (90.9% and 78.7% sensitivity; 75.3% and 79.4% specificity males and females, respectively) and high cardiovascular risk (63.2% and 50.0% sensitivity; 72.5% and 55.9% specificity for males and females, respectively) (Table 3).

## DISCUSSION

In this study, the NC and WHtR predictive capacity to diagnose obesity in Brazilian adolescents was evaluated, using the %BF obtained by ADP, and also to predict cardiovascular risk by means of PDAY. The NC cut-off points were 44.0 cm and 40.0 cm for males and females, respectively, and the WHtR cut-off point was 0.50 for both sexes.

The main result observed was the possibility of detecting obesity in adolescents of both sexes using the WHtR and NC. WHtR, especially, performed well when compared to NC, which performed reasonably well. In addition, to predict early cardiovascular risk, the two indices showed a reasonable ability for males, while both showed lower abilities for females.

According to BMI, the obesity prevalence among adolescents was observed only in males. This anthropometric index is considered better to discriminate excess body fat in males than in females^21^. Despite this, this prevalence was lower than that described in the national literature for this stage of life, which is around 8.4%^8^. Nevertheless, through ADP, higher prevalence was noted: 7.6% males and 39.4% females were obese.

Using anthropometry and anthropometric indicators in the obesity assessment is simple, fast, and inexpensive, and can be applied to a large number of individuals. In addition, BMI is the most used and recommended by the WHO to assess the adolescents’ nutritional status as well, but it is not capable of measuring or differentiating lean mass and fat mass as other methods (ADP, for example) do^30,31^.

Therefore, new indicators have been proposed for the prediction of central adiposity and, consequently, related to cardiovascular risk, including WHtR and NC, since they have been shown to be useful in the diagnosis of obesity in adolescents^32^.

With regard to the WHtR cut-off points defined for the analyzed adolescents, the cut-off point with the best diagnostic performance for obesity was 0.50, in both sexes, and presented an AUC of 0.87 and 0.90 for females and males, respectively, which is considered good/great. Most studies with adolescents indicate WHtR values equal to or close to 0.50. Dumith et al.^15^ identified 0.46 and 0.48 as cut-off points; Choi et al.^16^ referenced 0.50 and 0.48; Zhou et al.^10^determined 0.47 and 0.45; Marrodán et al.^17^, 0.51 and 0.50, for males and females, respectively; and Brannsether et al.^33^ reported 0.50 for both sexes. This cut-off point convergence identified in the studies makes WHtR safer and more valid as a good discriminator of obesity.

In this study, the NC cut-off points were 40 cm for females and 44 cm for males, and showed an obesity predictive capacity classified as moderate (♀ AUC 0.75 and 95%CI 0.69-0.80; ♂ AUC 0.70 and 95%CI 0.58-0.83). This measure had less variability described in national and international studies regarding the WHtR cut-off point for adolescents, as well as AUC, with values ≥ 0.80 (considered moderate/good) and sensitivity and specificity greater than 80%, which are considered good in terms of predictive performance.

The scientific discussion of these data obtained in the studies is complex, since the methods used to predict obesity from anthropometric indicators are different, as in international studies that determine the NC cut-off points for obesity or cardiovascular risk through the analysis of NC percentiles and not by the ROC curve^34,35^.

International studies have determined the NC cut-off points based on the ROC curve, such as that by Lou et al.^20^, which presents values from 27.4 to 31.3 cm for males and 26.3 to 31.4 cm for females; in turn, Hatipoglu et al.^36^ identified 32.5 cm and 31 cm in the pubertal phase for males and females, respectively. In those studies, children and adolescents were evaluated jointly and results showed AUC ≥ 0.75, sensitivity and specificity > 70% – predictive values considered good, corroborating the findings of this article.

In Brazil, there are few studies that determined predictive NC values in adolescence. Souza et al.^37^ evaluated a robust sample of adolescents aged 12 to 17 years (n = 1474) and the NC cut-off points performed well to identify obesity and cardiometabolic risk (between 15 and 17 years, the cut-off point was 38.4 cm for males and 35.8 cm for females, with AUC > 0.80 for both). Ferreti et al.^38^ evaluated 1,668 adolescents aged 10 to 17 years from public schools and identified the NC cut-off points, based on BMI, of 32.6 cm and 37.9 cm and AUC of 0.80 and 0.93 for males and females, respectively. Both studies used BMI to classify obesity.

Probably, the variation in the observed cut-off values is attributed to the difference in age groups, since this study evaluated adolescents aged 18 and 19 years, while the others evaluated a broader age group. Housseni et al.^35^ point out that there is a growing tendency for the increase in NC with age.

Another possible explanation for this variation would be the difference in the way of measuring NC and obesity in the studies. This study used the photonic scanner to measure NC, which do so from the three-dimensional body image, while in the cited studies an inelastic tape was used; and ADP to evaluate the %BF, while the others used less accurate methods, such as BMI, skinfolds and bioimpedance. Finally, the ethnic differences of the individuals evaluated in the studies are highlighted as an important factor.

With regard to cardiovascular risk, 12.2% adolescents in this study were classified as high risk according to PDAY, which, although not widespread, estimates the probability of early atherosclerotic lesions in adolescents and young adults, since it consists of an algorithm of global risk with multiple cardiovascular risk factors^22^. Among the risk factors included in the algorithm are the following: altered biochemical tests; high blood pressure; diagnosis of obesity, smoking; age, and sex.

However, all this information is not always accessible for the assessment of adolescents at high cardiovascular risk, and the identification of a simple, low-cost, and accessible anthropometric indicator for health care, such as NC and WHtR, could help in nutritional screening. In this study, to predict high cardiovascular risk, the same cut-off points were identified for NC, of 44 cm (AUC:0.71; 95%CI 0.62-0.80) for males and 40 cm (AUC:0.63; 95% CI0.53-0.73) for females, and for WHtR, of 0.50 for both sexes (♂AUC:0.73; 95%CI 0.63-0.82; and ♀ AUC:0.55, 95%CI 0.45-0.65).

In general, both anthropometric indicators showed good predictive capacity, but WHtR was not statistically significant to identify adolescents with high cardiovascular risk (lower limit of the 95%CI of AUC < 0.50).

Only one study was identified in the literature that evaluated the cardiovascular risk predictive capacity of NC using PDAY, and suggested the cutoff points of 35.6 cm for females and 36.6 cm for males in post-pubertal adolescents^26^. Furthermore, national and international studies – such as the one by Oliveira et al.^39^ – identify relationships between NC and WHtR only as isolated cardiovascular risk factors.

It is known that there is still no consensus on the anthropometric parameter that best correlates with metabolic changes and cardiovascular risk in adolescence. WHtR is considered simple to calculate and interpret, besides being an excellent non-invasive clinical screening tool^40^, recognized for having a strong correlation with cardiovascular outcomes and mortality^40^; in this study, however, it was not significant to detect cardiovascular risks in female adolescents.

NC is a relatively new indicator and, although more studies are needed to propose the identification of its cut-off points, it is considered a good predictor of obesity in children and adolescents^18,22^, as well as of metabolic risk factors and cardiovascular diseases^41^. Because it is not influenced by postprandial abdominal distension or respiratory movements^18^, it becomes advantageous in the service performance and was a good predictor of the outcomes listed in the study.

The fact that the study was consisted of a non-random subsample can be considered a limitation. However, the size of the subsample obtained does not differ from the studies available in the literature, and it is sometimes larger than some of these. In turn, the following positive aspects are listed: use of ADP, a method considered equivalent to the gold standard to identify obesity; the fact that it was the first Brazilian study to determine NC cut-off points to predict obesity in adolescents using ADP, and the use of PDAY to assess early cardiovascular risk, a global risk algorithm with multiple cardiovascular risk factors.

## CONCLUSION

There was high obesity prevalence in adolescents, especially in females, when evaluated by the %BF using PDA, a highly accurate method. NC of 40 cm and 44 cm for females and males, respectively, and WHtR of 0.50 for both sexes were determined as cut-off points to detect obesity and high cardiovascular risk.

The results of this study highlight WHtR and NC as good discriminators to assess obesity and cardiovascular risk in adolescents, especially males. Nevertheless, WHtR was limited in predicting cardiovascular risk in adolescents.

The study contributed by proposing WHtR and NC cut-off points as capable of predicting obesity and cardiovascular risk in adolescents, helping to screen these clinical conditions early, simply, and with low cost; besides, it can be used in the health promotion and health care services.
