# Supplementary material for: Appropriate neck circumference and waist-to-height ratio cut-off points as predictors of obesity and cardiovascular risk in adolescents
Source: Rev Saude Publica. 2023 Mar 30;57:24. doi: 10.11606/s1518-8787.2023057004349 (PMC10118407; doi:10.11606/s1518-8787.2023057004349)
Supplement: Translation

# Translation: Pontos de corte da circunferência do pescoço e da relação cintura-estatura como preditores da obesidade e risco cardiovascular em adolescentes

**Authors:** Wyllyane Rayana Chaves Carvalho, Ana Karina Teixeira da Cunha França, Alcione Miranda dos Santos, Luana Lopes Padilha, Eduarda Gomes Bogea

**Affiliations:** I Brasil Universidade Federal do Maranhão. Programa de Pós Graduação em Saúde Coletiva. São Luís, MA, Brasil; II Brasil Universidade Federal do Maranhão. Departamento de Saúde Pública. São Luís, MA, Brasil

**Keywords:** Adolescente, Razão Cintura-Estatura, Pescoço, Antropometria, Fatores de Risco de Doenças Cardíacas, Obesidade

## Abstract

**OBJETIVO:**

Determinar os pontos de corte da circunferência do pescoço (CP) e da relação cintura-estatura (RCEst) para a predição da obesidade e do risco cardiovascular em adolescentes.

**MÉTODOS:**

Estudo transversal desenvolvido com uma subamostra de 634 adolescentes de 18 e 19 anos de idade pertencentes à terceira fase da coorte “RPS” (Ribeirão Preto, Pelotas e São Luís) realizada em 2016. Identificou-se a área sob a curva ROC (AUC) para avaliar a capacidade preditiva da CP e RCE em relação ao percentual de gordura corporal (%GC), obtido pela pletismografia por deslocamento de ar (PDA), e do risco cardiovascular estimado pelo Phatobiological Determinants of Atherosclerosis in Youth (PDAY).

**RESULTADOS:**

A prevalência de obesidade pelo %GC foi de 7,6% no sexo masculino e 39,4% no sexo feminino (p-valor < 0,001) e o alto risco para PDAY foi de 13,8% e 10,9%, respectivamente. Para a CP, o ponto de corte identificado para o sexo masculino foi de 44,0 cm e as AUC foram de 0,70 (IC95% 0,58-0,83) para predição de obesidade e de 0,71 (IC95% 0,62-0,80) para predição do alto risco cardiovascular; e para o sexo feminino foi de 40 cm e as AUC foram de 0,75 (IC95% 0,69-0,80) e de 0,63 (IC95% 0,53-0,73), respectivamente. Para a RCEst, o ponto de corte identificado foi de 0,50 para ambos os sexos e as AUC para a predição da obesidade e do alto risco segundo o PDAY foram de 0,90 (IC95% 0,80-0,99) e 0,73 (IC95% 0,63-0,82), respectivamente, para o sexo masculino; e de 0,87 (IC95% 0,83-0,90) e 0,55 (IC95% 0,45-0,65), respectivamente, para o sexo feminino.

**CONCLUSÃO:**

RCEst e CP como bons discriminadores para avaliar a obesidade e risco cardiovascular em adolescentes, especialmente no sexo masculino.

## INTRODUÇÃO

Nas últimas décadas, o sobrepeso e a obesidade têm apresentado um crescimento substancial, sendo considerados desfechos preocupantes para a saúde pública em todo o mundo^1^. Destaca-se que os períodos mais críticos para o desenvolvimento do excedente de gordura corporal ocorrem na primeira infância e adolescência; entretanto, o acúmulo de gordura na adolescência tende a permanecer na fase adulta^2^.

É consenso na literatura que a obesidade, sobretudo a central, predispõe os indivíduos a doenças crônicas não transmissíveis (DCNT), o que pode ser explicado por relacionar-se a condições metabólicas específicas que favorecem a ocorrência de dislipidemias, hipertensão arterial, resistência à insulina e diabetes^3,4^.

A incidência dessas doenças tornou-se uma epidemia mundial^5^. Diante deste desafio, a Organização Mundial da Saúde (OMS) destaca como meta global para 2025 que se reduza a mortalidade prematura por DCNT em 25% e, para isto, um dos eixos estabelecidos é deter o aumento da prevalência de obesidade^6^. Nessa perspectiva, é importante a realização do diagnóstico da obesidade a partir da utilização de instrumentos simples e precisos para avaliação do excesso de gordura corporal.

Indicadores antropométricos são propostos para determinar a associação entre obesidade e risco cardiovascular em diversos estudos. A maior parte utilizou indicadores e métodos tradicionais, entre eles Índice de Massa Corporal (IMC) e Circunferência da Cintura (CC), a fim de comparar o desempenho desses indicadores na detecção de gordura corporal geral e do risco cardiovascular^7-9^.

Recentemente, outros indicadores começaram a ser estudados, entre eles a relação cintura-estatura (RCEst) e circunferência do pescoço (CP)^9^. A RCEst é considerada uma importante ferramenta para identificação da gordura corporal e risco de doenças cardiovasculares^5,13,14^, e tem ganhado destaque em estudos populacionais em diferentes faixas etárias^10,11^. Além disso, quando comparado a outros indicadores, sugere-se como vantagem a determinação de um único valor de ponto de corte da RCEst como um bom indicador antropométrico em saúde pública^12^. Em estudos nacionais e internacionais, observa-se uma pequena variação de pontos de corte da RCEst com objetivos de predizer a obesidade e risco cardiovascular em crianças, adolescentes e adultos, embora os valores determinados sejam próximos ou iguais a 0,50^9,14-17^.

No que se refere à CP, destaca-se que a gordura acumulada no pescoço é essencialmente subcutânea^7^, o que justificaria sua correlação com risco cardiovascular e resistência à insulina^18^, pois há uma maior atividade lipolítica desse compartimento de gordura, especialmente em indivíduos obesos^19^. Apesar de novo, este indicador apresenta como vantagens: ter um bom desempenho em determinar obesidade na infância e adolescência; ser aferido de maneira rápida e simples^7^; e não ser influenciada pela distensão abdominal pós-prandial ou por movimentos respiratórios^20^. No entanto, poucos são os estudos que avaliaram sua capacidade preditiva para obesidade e risco cardiovascular, especialmente em adolescentes.

Considerando a importância de identificar a capacidade preditiva, a partir de métodos considerados padrão-ouro, de indicadores antropométricos para a identificação de obesidade e risco cardiovascular em adolescentes e que possam ser utilizados na assistência à saúde, este estudo teve como objetivo determinar os pontos de corte da CP e da RCEst, a partir da pletismografia por deslocamento de ar (PDA), para a predição da obesidade e do risco cardiovascular em adolescentes.

## MÉTODOS

### Delineamento e Amostra do Estudo

Estudo transversal realizado com dados do Consórcio de Coortes de Nascimento RPS produzido por três cidades brasileiras (Ribeirão Preto, Pelotas e São Luís) e aprovado pelo Comitê de Ética em Pesquisa do Hospital Universitário da Universidade Federal do Maranhão (CEP/HU-UFMA) (Parecer Nº 1.302.489).

Os participantes da coorte da cidade de São Luís foram avaliados em três fases da vida: 1ª fase – nascimento; 2ª fase – infância (7 a 9 anos) e 3ª fase – adolescência (18 e 19 anos). A metodologia detalhada encontra-se no estudo de Bragança et al^21^. Para este trabalho utilizaram-se dados apenas da 3ª fase.

A 3ª fase da coorte foi realizada em 2016, com os indivíduos entre 18 e 19 anos de idade, e objetivou avaliar os desfechos nutricionais, doenças crônicas, saúde mental e capital humano. Nesta fase foram avaliados 2.515 adolescentes, sendo 654 pertencentes da fase do nascimento (1ª fase) e 1861 adolescentes nascidos em São Luís, MA, em 1997 que foram incluídos com objetivo de aumentar o poder da amostra e prever perdas futuras à coorte. Estes adolescentes incoporados na 3ª fase foram incluídos a partir da seleção nas quatro Juntas de Alistamento Militar na ilha de São Luís (MA); nas escolas de ensino médio com base em dados do censo escolar de 2014; e nas universidades. Ressalta-se que esses adolescentes foram submetidos aos mesmos testes e instrumentos aplicados aos adolescentes da fase de nascimento. Também foi aplicado um questionário às mães para coletar dados perinatais.

Destes 2.515 adolescentes avaliados na 3ª fase, foram incluídos neste estudo somente aqueles que tiveram dados da CP, CC e (%GC) aferidos. Após a aplicação destes critérios, 634 adolescentes foram elegíveis para compor a amostra final. A Figura 1 ilustra o plano amostral do estudo.

**Figura 1:** Fluxograma da amostra em estudo.

Sinasc: Sistema de Informação sobre Nascidos Vivos; CP: circunferência do pescoço.

### Coleta e Análise de Dados

Os dados foram obtidos por uma equipe treinada, por meio da aplicação de questionários e utilização de equipamentos, e registrados no programa *Research Eletronic Data Capture* (Redcap®)^22^.

Utilizaram-se os seguintes dados socioeconômicos e demográficos: sexo (masculino e feminino); idade (18 e 19 anos); classe socioeconômica, segundo Classificação Econômica no Brasil (CEB) (A/B, C e D/E); se estuda atualmente (sim e não); se trabalha atualmente (sim e não); cor da pele autodeclarada (preta, parda e branca); escolaridade (ensino fundamental, ensino médio, curso técnico nível médio, curso técnico profissionalizante, superior em andamento, curso pré-vestibular, EJA/PEJA); e hábito de fumar (sim e não).

Os dados antropométricos de interesse foram peso corporal, estatura, CC e CP. Para aferição do peso (em kg) utilizou-se a balança do próprio PDA, na qual os adolescentes foram posicionados em pé, no centro do equipamento, descalços, usando roupas de lycra justa ao corpo, sendo “top” para as mulheres e bermuda curta para ambos os sexos. A altura (cm) foi mensurada através do estadiômetro da marca Alturexata®, com os adolescentes descalços e em pé no centro do equipamento, com mãos ao longo do corpo, postura ereta, face voltada para frente, no Plano de Frankfurt, e observando um ponto fixo. A CP e CC (em cm) foram obtidas a partir da imagem tridimensional do corpo por meio do aparelho *3-Dimensional Photonic Scanner (3DPS-(*[TC] Labs, Cary, Estados Unidos). A relação cintura-estatura foi calculada por meio da razão entre CC e estatura.

Para avaliar a adequação do peso para a estatura foi utilizado o IMC. Este foi obtido por meio da razão entre peso corporal (kg) e estatura (m^2^), classificado em escore-Z, de acordo com sexo e idade. Foram utilizados os critérios: baixo peso (< Escores-Z -2); eutrofia (≥ Escores-Z -2 e < Escores-Z +1); sobrepeso (≥ Escore-Z +1 e < Escore-Z +2) e obesidade (≥ Escore-Z +2)^23^. Para a avaliação da gordura abdominal foi utilizada a CP e RCEst.

Para a verificação da adiposidade corporal foi utilizada a técnica da PDA, por meio do aparelho Bod Pod*®* Gold Standard da marca Cosmed (Roma, Itália). No momento do teste, os adolescentes estavam com as mesmas vestimentas utilizadas na aferição das medidas antropométricas e foi fornecida uma touca para comprimir os cabelos durante a PDA. O pletismógrafo era calibrado diariamente com um volume conhecido de 50 litros. Com base no volume corporal mensurado e da massa corporal, o aparelho calculava a densidade corporal, que foi utilizada na equação de Siri para determinar a massa de gordura dos adolescentes^24^. O percentual de gordura corporal dos adolescentes foi classificado em obeso (≥ 25% para homens e ≥ 30% para mulheres) e não obeso (< 25% para homens e < 30% para mulheres) por Williams et al^25^.

A pressão arterial foi verificada pelo método oscilométrico com aparelho digital Omron® modelo HEM-7221NT. Foram utilizados manguitos de tamanho apropriado à circunferência dos braços e considerada a média das três medidas de pressão arterial sistólica e diastólica aferidas, após um minuto de descanso, na posição sentada, com o braço dominante apoiado em suporte de modo que a artéria radial ficasse no mesmo nível do coração.

Os marcadores bioquímicos utilizados foram glicemia pós-prandial, colesterol total, HDL e LDL, mensurados a partir da análise do sangue dos indivíduos. Realizou-se a separação do soro e armazenamento em *eppendorf* a -80°C até o momento da análise. No momento da coleta de sangue os adolescentes não estavam em jejum e não foram questionados sobre o horário de sua última refeição realizada. As amostras foram analisadas no laboratório da Faculdade de Odontologia da Universidade Federal do Maranhão (UFM), por meio do Milliplex MAP Human Cytokine Kit, fabricado pela Merck (Darmestádio, Alemanha).

O risco cardiovascular foi avaliado por meio do *Pathobiological Determinants of Atherosclerosis in Youth* (PDAY), que é um algoritmo de risco global com múltiplos fatores de risco cardiovascular, e apresenta como vantagem estimar a probabilidade de lesões ateroscleróticas precoces em adolescentes e adultos jovens^26^. Este foi desenvolvido com base no Framingham Risk Score (FRS) e estabelece como premissa que fatores de risco para DCV estão associados, décadas antes do desfecho cardiovascular, com as fases inicial e avançada das lesões ateroscleróticas durante a adolescência e início da vida adulta. A estratificação do risco pelo PDAY é obtida pela soma dos valores atribuídos a fatores modificáveis como colesterol não HDL, colesterol HDL, tabagismo, pressão arterial, IMC, glicemia de jejum (GJ) e hemoglobina glicosilada HBA1c, bem como fatores demográficos (idade, sexo). Se o resultado do somatório obtido for superior a zero estima-se probabilidade para lesões ateroscleróticas; portanto, risco cardiovascular^27,28^.

Assim, considerando estas estratificações, o PDAY foi obtido neste estudo a partir das variáveis e suas respectivas pontuações: idade (em anos, de 10-19 = 0; 20-24 = 5; 25-29 = 10; 30-34 = 15 pontos), sexo (masculino = 0; feminino = -1 ponto), colesterol não HDL (em mg/dL, < 130 = 0; 130-159 = 2; 160-189 = 4; 190-219 = 6; ≥ 220 = 8 pontos), HDL (em mg/dL, < 40 = 1; 40-59 = 0; ≥ 60 = -1 ponto), tabagismo (não = 0; sim = 1 ponto), pressão arterial (normal = 0; elevada = 4), obesidade (avaliada pelo IMC, não obeso = 0 e obeso = 6 para o sexo masculino; não obeso e obeso = 0 para o sexo feminino) e hiperglicemia (glicose pós prandial < 140mg/dL = 0 e glicose em jejum ≥ 140mg/dL = 5 pontos). A partir do somatório das pontuações de cada variável, o risco cardiovascular foi classificado em baixo (pontuação = 0), intermediário (pontuação ≥ 1 e ≤ 4) e alto (pontuação ≥ 5 pontos).

### Análise Estatística

As variáveis sociodemográficas, nutricionais e de risco cardiovascular foram descritas por meio de frequências absolutas e relativas. Apenas a variável idade foi descrita por meio de média e desvio padrão. Para verificação da normalidade da variável idade foi calculado o coeficiente de assimetria, de curtose e o teste Shapiro-Wilk.

Utilizou-se a curva ROC (*receiver-operator curve*) para analisar a validade preditiva da CP e RCEst em discriminar os adolescentes obesos, em relação ao %GC obtido pela PDA, e com alto risco cardiovascular, em relação ao PDAY. Foram determinadas as áreas sob a curva (AUC) ROC e os intervalos de confiança, e identificados os valores da CP e RCEst com melhor equilíbrio entre sensibilidade e especificidade.

A curva ROC é um método gráfico para avaliação, organização e seleção de sistemas de diagnóstico e/ou predição. A AUC descreve a probabilidade de identificar corretamente indivíduos que são verdadeiro-positivos e aqueles que não são. Estes valores apresentam significância estatística quando o limite inferior do IC95% for superior a 0,50. São considerados valores da área sob a curva ROC excelentes quando entre 0,90–1,00; bons, entre 0,80–0,90; razoáveis, entre 0,70–0,80; e pobres, entre 0,60–0,70^29^.

Os dados foram exportados do sistema REDCap para análise no programa STATA® (versão 14.0). Foram adotados nível de significância de 5% e intervalo de confiança de 95% (IC95%).

## RESULTADOS

Foram avaliados 634 adolescentes: a média de idade foi de 18,5±0,5 anos e a maioria era do sexo feminino (54,4%), solteiros (98,0%), autodeclarados pardos (61,4%) e pertencentes à classe C (43,7%); 35,2% referiram cursar o pré-vestibular ou estar cursando ensino superior (Tabela 1).

**Tabela 1:** Características socioeconômicas e demográficas de adolescentes da coorte de nascimento RPS (Terceira fase), São Luís, Maranhão, Brasil, 2016.

Variáveis	n	%
Sexo		
Masculino	289	45,6
Feminino	345	54,4
Estado civil		
Solteiro	621	98,0
União estável	13	2,0
Cor		
Branca	133	21,0
Preta	106	16,7
Parda/mulata/cabocla/ Morena	389	61,4
Amarela/oriental	2	0,3
Ignorado	4	0,6
Escolaridade		
Fundamental	1	0,2
Curso técnico ou profissionalizante	30	4,7
Ensino superior em andamento	223	35,2
Curso pré vestibular	223	35,2
EJA/PEJA^a^	11	1,7
Ignorado	146	23,0
Classe econômica^b^		
A/B	203	32,0
C	277	43,7
D/E	98	15,5
Ignorado	56	8,8
Total	634	100,0

^a^ EJA/PEJA: Educação de Jovens e Adultos/Programa de Educação de Jovens e Adultos.

^b^ De acordo com o Critério de Classificação Econômica Brasil-ABEP.

Notou-se obesidade, segundo o diagnóstico do IMC, apenas no sexo masculino: 3,8%. Por meio da PDA, observou-se prevalência de obesidade de 7,6% no sexo masculino e 39,4% no sexo feminino (p-valor < 0,001). A prevalência de adolescentes com alto risco cardiovascular, avaliado pelo PDAY, foi maior no sexo masculino (13,8% *versus* 10,9%; p < 0,001) (Tabela 2).

**Tabela 2:** Estado nutricional e risco cardiovascular de adolescentes da coorte de nascimento RPS (Terceira fase), São Luís, Maranhão, Brasil, 2016.

Variáveis	Total n (%)	Homens n (%)	Mulheres n (%)	p-valor
IMC				0,001
Baixo	26 (4,1)	10 (3,5)	16 (4,6)	
Eutrofia	505 (79,7)	235 (81,3)	270 (78,3)	
Sobrepeso	92 (14,5)	33 (11,4)	59 (17,1)	
Obesidade	11 (1,7)	11 (3,8)	0 (0,0)	
%GC, por PDA				< 0,001
Não obeso	476 (75,1)	267 (92,4)	209 (60,6)	
Obeso	158 (24,9)	22 (7,6)	136 (39,4)	
Risco cardiovascular, pelo PDAY				< 0,001
Baixo	398 (61,4)	149 (51,4)	249 (69,6)	
Intermerdiário	171 (26,4)	101 (34,8)	70 (19,6)	
Alto	79 (12,2)	40 (13,8)	39 (10,9)	

IMC: índice de massa corporal. %GC: percentual de gordura corporal. PDA: pletismografia por deslocamento de ar. PDAY: *Pathobiological Determinants of Atherosclerosis in Youth*.

A AUC-ROC da CP e RCEst para a predição da obesidade são apresentadas na Figura 1 e Tabela 3. Para o sexo masculino, a AUC da CP foi de 0,70 (IC95% 0,58–0,83) e da RCEst, 0,90 (IC95% 0,80–0,99), enquanto para o sexo feminino a AUC da CP foi 0,75 (IC95% 0,69–0,80) e da RCEst, 0,87 (IC95% 0,83–0,90). Os indicadores antropométricos avaliados apresentaram capacidade preditiva estatisticamente significante para identificar indivíduos obesos em ambos os sexos.

**Tabela 3:** Sensibilidade e especificidade dos pontos de corte da CP e RCEst em relação à medida de adiposidade corporal elevada obtida pelo PDA, e do risco cardiovascular avaliado pelo PDAY, de adolescentes da coorte de nascimento RPS (terceira fase), São Luís, Maranhão, Brasil, 2016.

Variáveis	AUC	IC95%	PC	Sensibilidade	Especificidade
**Sexo masculino**					
%GC					
CP (cm)	0,70	0,58-0,83	44,0	68,2%	65,9%
RCEst	0,90	0,80-0,99	0,50	90,9%	75,3%
PDAY					
CP (cm)	0,71	0,62-0,80	44,0	63,2%	63,8%
RCEst	0,73	0,63-0,82	0,50	63,2%	72,5%
**Sexo feminino**					
%GC					
CP (cm)	0,75	0,69- 0,80	40,0	64,7%	72,2%
RCEst	0,87	0,83-0.90	0,50	78,7%	79,4%
PDAY					
CP (cm)	0,63	0,53-0,73	40,0	61,8%	59,2%
RCEst	0,55	0,45-0,65	0,50	50,0%	55,9%

PDA: pletismografia por deslocamento de ar. %GC: percentual de gordura corporal. AUC: área sob a curva Roc. IC: intervalo de confiança. CP: circunferência do pescoço. RCE: relação cintura-estatura. PDAY: *Pathobiological Determinants of Atherosclerosis in Youth.*

Na Figura 3 e Tabela 3 são apresentadas a AUC-ROC da CP e RCEst para a predição do alto risco cardiovascular. Para o sexo masculino, a AUC da CP foi de 0,71 (IC95% 0,62–0,80) e da RCEst, 0,73 (IC95% 0,63–0,82), enquanto para o sexo feminino a AUC da CP foi de 0,63 (IC95% 0,53–0,73) e da RCEst, 0,55 (IC95% 0,45–0,65). Apenas a RCEst não apresentou capacidade preditiva estatisticamente significante para identificar as adolescentes com alto risco cardiovascular.

**Figura 3:** Área sob a curva Roc e IC95% da CP e RCEst com risco cardiovascular, avaliada pelo *Pathobiological Determinants of Atherosclerosis in Youth* (PDAY), em adolescentes de ambos os sexos da coorte de nascimento RPS (terceira fase), São Luís, Maranhão, Brasil, 2016.

Para a predição da obesidade e do alto risco cardiovascular foram identificados os pontos de corte da CP de 40,0 cm para o sexo feminino (sensibilidade e especificidade de 64,7% e 72,2% para obesidade, respectivamente; e 61,8% e 59,2%, para o risco cardiovascular, respectivamente) e 44,0 cm para o sexo masculino (68,2% de sensibilidade e 65,9% de especificidade para a obesidade; e 63,2% de sensibilidade e 63,8% de especificidade para o risco cardiovascular). Para a RCEst, foi identificado o ponto de corte de 0,50 em ambos os sexos tanto para predizer a obesidade (sensibilidade = 90,9% e 78,7%; especificidade = 75,3% e 79,4%, para o sexo masculino e feminino, respectivamente) quanto o alto risco cardiovascular (sensibilidade = 63,2% e 50,0%; especificidade = 72,5% e 55,9%, para o sexo masculino e feminino, respectivamente) (Tabela 3).

## DISCUSSÃO

Neste estudo, avaliou-se a capacidade preditiva da CP e RCEst no diagnóstico da obesidade em adolescentes brasileiros, utilizando o %GC obtido pela PDA, bem como na predição do risco cardiovascular por meio do PDAY. Os pontos de corte definidos para a CP foram 44,0 cm e 40,0 cm para os sexos masculino e feminino, respectivamente, e para a RCEst 0,50 em ambos os sexos.

O principal resultado observado foi a possibilidade de detecção da obesidade, em adolescentes de ambos os sexos, por meio da relação cintura-estatura e da circunferência do pescoço. A RCEst, especialmente, teve bom desempenho quando comparada à CP, que apresentou desempenho razoável. Além disso, para a predição do risco cardiovascular precoce, os dois índices apresentaram uma capacidade razoável para o sexo masculino, enquanto ambos apresentaram capacidades inferiores para o sexo feminino.

A prevalência de obesidade entre os adolescentes, pelo IMC, foi observada apenas no sexo masculino. Este índice antropométrico é considerado melhor para discriminar excesso de gordura corporal em homens do que em mulheres^21^. Apesar disso, esta prevalência foi menor do que a descrita pela literatura nacional para essa fase da vida, que é em torno de 8,4%^8^. Entretanto, por meio da PDA notaram-se prevalências maiores: 7,6% dos meninos e 39,4% das meninas eram obesos.

É válido destacar que o uso da antropometria e de indicadores antropométricos na avaliação da obesidade são simples, rápidos, de baixo custo e podem ser aplicados em um grande número de indivíduos. Além disso, o IMC é o mais utilizado e recomendado pela OMS para avaliação do estado nutricional também em adolescentes, mas não é capaz de mensurar nem diferenciar massa magra e massa gorda como outros métodos (a PDA, por exemplo) fazem^30,31^.

Para tanto, novos indicadores foram propostos para a predição da adiposidade central e, consequentemente, relacionados ao risco cardiovascular, entre eles a RCEst e a CP, uma vez que têm se apresentado úteis no diagnóstico da obesidade em adolescentes^32^.

No que diz respeito aos pontos de corte definidos para a RCEst dos pesquisados, o ponto de corte com melhor desempenho diagnóstico para obesidade foi de 0,50, em ambos os sexos, e apresentou AUC de 0,87 e 0,90 para o sexo feminino e masculino, respectivamente, considerados bons/ótimos. Grande parte dos estudos com adolescentes indicam valores de RCEst iguais ou próximos a 0,50. Dumith et al.^15^ identificaram 0,46 e 0,48 como pontos de corte; Choi et al.^16^ referenciaram 0,50 e 0,48; Zhou et al.^10^ determinaram 0,47 e 0,45; Marrodán et al.^17^, 0,51 e 0,50, para meninos e meninas, respectivamente; e Brannsether et al.^33^ referiram 0,50, para ambos os sexos. Essa convergência dos pontos de corte identificados nos estudos o torna mais seguro e válido como um bom discriminador da obesidade.

Neste estudo, para a CP os pontos de corte foram 40 cm para o sexo feminino e 44 cm para o masculino, e apresentaram capacidade preditiva para a obesidade classificada como moderada (♀ AUC 0,75 e IC95% 0,69-0,80; ♂ AUC 0,70 e IC95% 0,58-0,83). Salienta-se, também, que essa medida apresentou uma menor variabilidade descrita nos estudos nacionais e internacionais quanto ao ponto de corte da RCEst para adolescentes, bem como a AUC, notando-se valores ≥ 0,80 (consideradas moderada/boa) e sensibilidade e especificidade superior a 80%, que são considerados bons quanto ao desempenho preditivo.

É complexa a discussão científica desses dados obtidos nos estudos, pois os métodos utilizados para predição da obesidade de indicadores antropométricos são distintos, a exemplo de estudos internacionais que determinam os pontos de corte da CP para obesidade ou risco cardiovascular por meio da análise dos percentis da CP e não pela curva Roc^34,35^.

Os estudos internacionais determinaram os pontos de corte da CP a partir da curva Roc, como o de Lou et al.^20^ que apresenta valores de 27,4 a 31,3 cm para meninos e 26,3 a 31,4 cm para meninas; Hatipoglu et al.^36^ identificaram 32,5 cm e 31 cm na fase puberal para meninos e meninas, respectivamente. Estes estudos avaliaram conjuntamente crianças e adolescentes e apresentaram AUC ≥ 0,75, sensibilidade e especificidade > 70% – valores preditivos considerados bons, corroborando os achados deste artigo.

No Brasil, são escassos os trabalhos que determinaram valores preditivos da CP na adolescência. Souza et al.^37^ avaliaram uma amostra robusta de adolescentes de 12 a 17 anos (n = 1474) e os pontos de corte da CP apresentaram bom desempenho para identificar a obesidade e risco cardiometabólico (entre 15 e 17 anos, o ponto de corte foi 38,4 cm para o sexo masculino e 35,8 cm para o feminino, com AUC > 0,80 em ambos). Ferreti et al.^38^ avaliaram 1.668 adolescentes de 10 a 17 anos de escolas públicas e identificaram os pontos de corte da CP, a partir do IMC, de 32,6 cm e 37,9 cm e as AUC de 0,80 e 0,93 para meninos e meninas, respectivamente. Ambos os estudos utilizaram o IMC para classificação da obesidade.

É provável que a variação dos valores de ponto de corte observados seja atribuída à diferença da faixa etária, uma vez que avaliamos adolescentes com idades de 18 e 19 anos, enquanto os demais avaliaram uma faixa etária mais ampla. Housseni et al.^35^ressalvam que há uma tendência crescente do aumento da CP com a idade.

Outra possível explicação para essa variação seria a diferença na forma de mensurar a CP e a obesidade nas pesquisas. Utilizamos o aparelho *Photonic* para mensuração das medidas de circunferências, que afere a medida a partir da imagem tridimensional do corpo, enquanto nos estudos citados foi utilizada a fita inelástica; e o PDA para avaliação do %GC, enquanto os demais utilizaram métodos menos acurados, tais como o IMC, dobras cutâneas e bioimpedância. Por fim, salienta-se como um fator importante as diferenças étnicas dos indivíduos avaliados nos estudos.

No que diz respeito ao risco cardiovascular, 12,2% dos adolescentes neste estudo foram classificados como de alto risco segundo PDAY que, apesar de pouco difundido, estima a probabilidade de lesões ateroscleróticas precoces em adolescentes e adultos jovens, pois consiste em um algoritmo de risco global com múltiplos fatores de risco cardiovascular^22^. Entre os fatores de risco inseridos no algoritmo estão: exames bioquímicos alterados, pressão arterial elevada, diagnóstico de obesidade, hábito de fumar, idade e sexo.

Entretanto, nem sempre são acessíveis todas estas informações para avaliação de adolescentes com alto risco cardiovascular, e a identificação de um indicador antropométrico simples, de baixo custo e acessível à assistência à saúde, como a CP e a RCEst, poderiam auxiliar na triagem nutricional. Neste estudo, para a predição do alto risco cardiovascular, foram identificados os mesmos pontos de corte para a CP de 44 cm (AUC:0,71; IC95% 0,62-0,80) para o sexo masculino e de 40 cm (AUC:0,63; IC95% 0,53-0,73) para o feminino, e para RCEst de 0,50 para ambos os sexos (♂ AUC:0,73; IC95% 0,63-0,82; e ♀ AUC:0,55, IC95% 0,45-0,65).

De uma forma geral, ambos os indicadores antropométricos apresentaram boa capacidade preditiva, porém a RCEst não se apresentou estatisticamente significante para identificar as adolescentes com alto risco cardiovascular (limite inferior do IC95% da AUC < 0,50).

Identificou-se na literatura apenas um estudo que avaliou a capacidade preditiva da CP para risco cardiovascular utilizando o PDAY, e sugeriu os pontos de corte de 35,6 cm para o sexo feminino e 36,6 cm no masculino nos adolescentes na fase pós puberal^26^. Ademais, os estudos nacionais e internacionais – como o de Oliveira et al.^39^ – identificam relações da CP e RCEst apenas como fatores de risco cardiovasculares isolados.

É sabido que ainda não há consenso sobre o parâmetro antropométrico que melhor se correlaciona a alterações metabólicas e risco cardiovascular na adolescência. A RCEst é considerada simples de calcular, interpretar e uma excelente ferramenta de triagem clínica não invasiva^40^, reconhecida por apresentar uma forte correlação com desfechos cardiovasculares e mortalidade^40^; neste estudo, entretanto, não apresentou significância para detectar riscos cardiovasculares em adolescentes do sexo feminino.

A CP é um indicador relativamente novo e, embora sejam necessários mais estudos que proponham a identificação de seus pontos de corte, é considerada um bom preditor da obesidade em crianças e adolescentes^18,22^, bem como para fatores de risco metabólicos e doenças cardiovasculares^41^. Por não ser influenciada pela distensão abdominal pós-prandial ou por movimentos respiratórios^18^, torna-se vantajosa na prática dos serviços e apresentou-se como bom preditor para os desfechos elencados no estudo.

Uma limitação do estudo foi ser constituído por uma subamostra não aleatória. Contudo, o tamanho da subamostra obtida não difere dos estudos dispostos na literatura, e é por vezes maior que alguns destes. Por sua vez, como aspectos positivos são elencados: utilização da PDA, método considerado equivalente ao padrão-ouro para a identificação da obesidade; o fato de ser o primeiro estudo brasileiro a determinar pontos de corte da CP na predição da obesidade em adolescentes utilizando a PDA; e a utilização do PDAY para avaliação do risco cardiovascular precoce, um algoritmo de risco global com múltiplos fatores de risco cardiovascular.

## CONCLUSÃO

Observou-se uma alta prevalência de obesidade nos adolescentes, especialmente no sexo feminino, quando avaliada pelo %GC por meio da PDA, método de elevada acurácia. Os valores para CP de 40 cm e 44 cm para os sexos feminino e masculino, respectivamente, e para a RCEst de 0,50 em ambos os sexos foram determinados como pontos de corte para a detecção da obesidade e de alto risco cardiovascular.

Os resultados deste estudo destacam a RCEst e CP como bons discriminadores para avaliar a obesidade e risco cardiovascular em adolescentes, especialmente do sexo masculino. No entanto, a RCEst apresentou limitação para predizer risco cardiovascular nas adolescentes.

O estudo contribuiu propondo pontos de corte da circunferência do pescoço e relação cintura-estatura como capazes de predizer obesidade e risco cardiovascular em adolescentes, auxiliando na triagem dessas manifestações clínicas de forma precoce, simples, de baixo custo e acessível na utilização nos serviços de promoção e assistência à saúde.

**Figura 2:** Área sob a curva Roc e IC95% da CP e RCEst com obesidade, avaliada pelo pletismografia por deslocamento de ar (PDA), em adolescentes de ambos os sexos da coorte de nascimento RPS (terceira fase), São Luís, Maranhão, Brasil, 2016.
